# Parent-Reported Burn-Specific Health-Related Quality of Life in Children 5–7 Years After Burns: A Multicenter Cross-Sectional Study

**DOI:** 10.3390/ebj6010005

**Published:** 2025-01-30

**Authors:** Marina C. Heijblom, J. Nicolaas Dijkshoorn, Marianne K. Nieuwenhuis, Anouk Pijpe, Cornelis H. van der Vlies, Margriet E. van Baar, Inge Spronk

**Affiliations:** 1 Burn Centre, Maasstad Hospital, 3079 DZ Rotterdam, The Netherlands; dijkshoornj@maasstadziekenhuis.nl (J.N.D.); vliesc@maasstadziekenhuis.nl (C.H.v.d.V.); 2 Alliance of Dutch Burn Centres, Maasstad Hospital, 3079 DZ Rotterdam, The Netherlands; baarm@maasstadziekenhuis.nl (M.E.v.B.); ispronk@burns.nl (I.S.); 3 Trauma Research Unit, Department of Surgery, Erasmus MC, University Medical Centre Rotterdam, 3015 GD Rotterdam, The Netherlands; 4 Alliance of Dutch Burn Care (ADBC), Burn Centre, Martini Hospital, 9728 NT Groningen, The Netherlands; nieuwemk@mzh.nl; 5 Research Group Healthy Ageing, Allied Health Care and Nursing, Hanze University of Applied Sciences, 9714 CA Groningen, The Netherlands; 6 Department for Human Movement Sciences, University Medical Center Groningen, University of Groningen, 9713 GZ Groningen, The Netherlands; 7 Alliance of Dutch Burn Care (ADBC), Burn Centre, Red Cross Hospital, 1942 LE Beverwijk, The Netherlands; apijpe@rkz.nl; 8 Department of Plastic, Reconstructive and Hand Surgery, Amsterdam UMC Location Vrije Universiteit Amsterdam, 1081 HV Amsterdam, The Netherlands; 9 Amsterdam Movement Sciences (AMS), Tissue Function and Regeneration, 1105 AZ Amsterdam, The Netherlands; 10 Department of Public Health, Erasmus MC, 3015 GD Rotterdam, The Netherlands; 11 Dutch Burns Foundation, 1941 AJ Beverwijk, The Netherlands

**Keywords:** burns, children, health-related quality of life, long-term assessment

## Abstract

Surviving a burn can dramatically alter a child’s life, yet few studies examined long-term health-related quality of life (HRQL). This study assessed HRQL 5–7 years post-burn in children with mild/intermediate and severe burns and identified associated factors. Parents of children (5− < 18 years) who were hospitalized or had burn surgery between 08/2011 and 09/2012 completed the Burn Outcomes Questionnaire (BOQ). Outcomes were compared between two subgroups: children with mild/intermediate burns (<10% total body surface area (TBSA) burned) versus severe burns ((1) aged <10 years old at the time of injury with >10% (TBSA) burned; (2) aged ≥10 years with >20% TBSA burned; or (3) >5% full-thickness burns). A total of 102 children were included (mean age at survey: 8.4 (3.0) years; mean former TBSA: 7.1%). At a mean of 5.7 years post-burn, many parents rated their child’s health as excellent (46.1%) or very good (35.3%), with few reporting issues with ‘pain’ (2.3%), ‘physical function and sports’ (1.6%), and ‘upper extremity function’ (0.9%). Parents of children with severe burns indicated significantly more problems with ‘appearance’ (89.2% versus 71.5%; *p* = 0.014) and ‘parental concern’ (94.1% versus 84.8%; *p* = 0.021). Upper limb burns, facial burns, burn size, length of hospital stay, full-thickness burns, and the number of surgeries predicted poorer outcomes. In general, these findings indicate positive long-term HRQL, though especially children with full-thickness burns and/or surgical interventions face a higher risk of reduced HRQL. The results can be used to inform children and their families about the long-term implications. Furthermore, healthcare professionals can use these insights to identify children at higher risk of poorer long-term HRQL.

## 1. Introduction

A child’s life can change dramatically when they survive a burn injury. In the Netherlands, 43% of all burns occur in children [1,2]. Worldwide, children are also a large part of the burn population [3]. Besides children themselves, burn injuries affect their parents and other family members who must cope with the circumstances and effects of their child’s burn injury [4]. Despite improved treatments, children have to deal with cosmetic disfigurement and physical and psychological problems shortly after the injury, but also in the longer term [5,6]. An outcome that captures a wide range of problems, including the physical, emotional, and social aspects of health, is health-related quality of life (HRQL) [4,5,7,8]. HRQL is a patient-reported outcome that measures an individual’s perceived well-being in relation to their health. This outcome is therefore increasingly used in burn care and research to evaluate children’s recovery from a burn injury [4].

Pediatric HRQL can be measured with a variety of questionnaires. Most of them are questionnaires that children, parents of children, or observers have to fill in [9]. The HRQL of children with burns is measured with a generic or a burn-specific HRQL instrument [10]. Generic instruments are designed to measure HRQL across a wide range of health conditions and populations and are not specific to any particular disease or condition; burn-specific instruments are specifically developed for children with burns and include aspects of quality of life that are most relevant to burn-specific health effects. In order to assess the full impact of burns, it is important to use a burn-specific questionnaire to measure HRQL in children with burns [11]. Multiple burn-specific pediatric HRQL instruments exist [10,12,13,14,15]. A commonly used instrument to measure HRQL in children is the Burn Outcomes Questionnaire (BOQ), which is considered a feasible, valid, and reliable instrument [16,17].

Studies on long-term (>5 years post-burn) HRQL in children with burns are scarce; most studies focus on shorter-term HRQL up to two years post-burn [10]. Earlier studies show that pediatric HRQL improves over time [10]. However, longer-term psychosocial problems, such as appearance-related issues, anxiety/depression, and parental concern, have been observed. The few studies that focused on the longer-term impact of burns mostly focused on children with severe burns [18,19,20,21]. Therefore, the aim of this study was to study the long-term parent-reported burn-specific HRQL and predictive factors of poorer HRQL in children with all burns in specialized burn care 5–7 years after their injury. The secondary aim of this study was to compare HRQL between children with mild/intermediate burns and those with severe burns.

## 2. Materials and Methods

This cross-sectional study was performed as part of the Burden of Burn Injuries project on the long-term consequences of burn injuries. It was approved by the Ethics Committee (number NL59981) and the institutional review boards of Maasstad Hospital Rotterdam, Martini Hospital Groningen, and Red Cross Hospital Beverwijk, and it was conducted following the principles of the Declaration of Helsinki. This study was registered with the Netherlands Trial Register (number NTR6407) [18]. Both parents of each child signed a written informed consent form. When a child was 12 years old or above, the child also signed the consent form.

Our study population included pediatric burn patients (5–17 years of age at the time of the survey) who had been hospitalized for ≥1 day or who had had surgery for their burns at a Dutch burn center (Maasstad Hospital Rotterdam, Martini Hospital Groningen, Red Cross Hospital Beverwijk) between August 2011 and September 2012. For the cohort of patients with severe burns, we included patients who were hospitalized in one of the three Dutch burn centers in the period January 2010–December 2013. Children with mild/intermediate burns were defined as having <10% TBSA burned. Children with severe burns were defined as (1) aged <10 years old at the time of injury with >10% (TBSA) burned; (2) aged ≥10 years old at the time of injury with >20% TBSA burned; or (3) having >5% full-thickness burns. The patients were identified from the Dutch Burn Repository R3 [4]. Pediatric patients were eligible for this study when they were not deceased, their parents were able to answer questionnaires in Dutch, and when contact information was available at the time of this study.

Parents of eligible pediatric patients 5–17 years old received an invitation letter, an information brochure, an informed consent form, and the first short survey about general physical and emotional functioning via post between March 2017 and March 2018. After receiving informed consent and the first survey, a second, more extensive survey including a burn-specific HRQL questionnaire (i.e., the Burn Outcomes Questionnaire (BOQ)) was sent by post or email, depending on the parent’s indicated preference. The second survey, including the BOQ, was sent as soon as the first survey with informed consent had been received by the research team. Surveys were completed by parents. If the first survey was not returned within three weeks, parents were called and invited to participate. If no phone number was available or parents could not be reached, a postal reminder was sent. For the second survey, a reminder was sent by post or email if no response was received after three weeks of sending the second survey.

### 2.1. Measures

Parent-reported pediatric burn-specific HRQL was assessed with the Dutch version of the Burn Outcomes Questionnaire version 5–18 years old (BOQ 5-18) [16,17]. This multidimensional instrument contains two questions on children’s health status and includes another 53 questions in 12 domains: upper extremity function, physical function and sports, transfers and mobility, itch, pain, compliance with treatment, satisfaction with current state, appearance, emotional health, parental concern, school re-entry, and family disruption. Domain scores were calculated by averaging the answers to the questions corresponding to that particular domain. If fewer than 50% of the questions for a particular domain were answered, then the scale was assigned to a missing value. Scoring for the domains pain, itch, family disruption, and parental concern was reversed so that higher scores indicated better outcomes for all domains. Scores for school re-entry should be interpreted differently. A score greater than 50 is the ideal score. The Dutch version of the BOQ 5-18, which was validated before, was used [16,17].

Patient, burn, and clinical characteristics were extracted from the Dutch Repository R3 [2]. Patient characteristics included age and sex. Burn characteristics included %TBSA burned, %TBSA full-thickness burns, etiology, location of burn injury, and date of burn. Clinical characteristics were length of hospital stay (LOS), number of surgeries, reconstructive surgery (defined as surgery after wound closure) (yes/no), and artificial ventilation (yes/no).

### 2.2. Statistical Analyses

The characteristics of participating and non-participating children were compared in a non-response analysis to investigate significant differences using descriptive statistics. Patient characteristics and HRQL outcomes of children were studied for the whole sample and compared between the two subgroups—children with mild/intermediate burns and children with severe burns—according to the definitions described above. Mann–Whitney U tests were used to compare continuous variables, and chi-square tests were used for categorical variables. In case of small numbers (<5), Fisher’s exact test was used.

To study the predictive factors of the HRQL domains, we performed univariate linear regression analyses on the overall sample. All factors with a *p*-value < 0.10 were tested for collinearity (>0.8 or <−0.8). Univariate predictors with a *p*-value < 0.10 were studied in an enter-method multivariate linear regression analysis. In case of missing values on the BOQ domains, patients were excluded from regression analyses. A *p*-value of <0.05 was considered statistically significant. IBM SPSS Statistics 26 was used for all analyses.

## 3. Results

### 3.1. Participants

**Figure 1:**
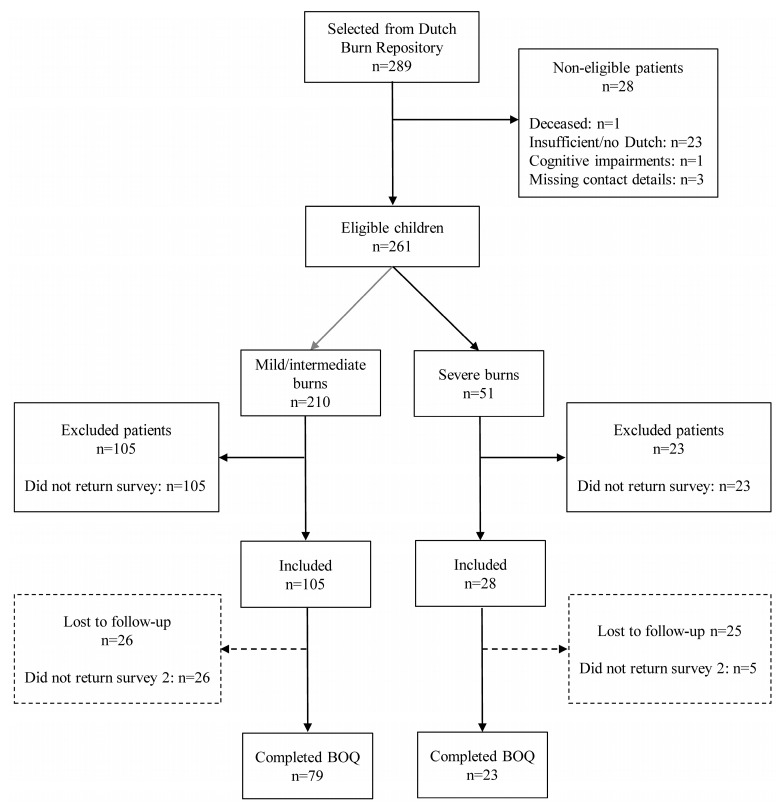
Flowchart on patient selection.

In total, 289 children were identified in the Dutch Burn Repository R3, of whom 261 were eligible for this study. Parents of 133 children (51.0%) completed the first survey and were willing to participate. Of these, 102 completed at least one question of the BOQ in the second survey and were therefore included in the present study (Figure 1). Thirty-one parents (11.8%) did not return the second survey.

Parents of 79 children with mild/intermediate burns and parents of 23 children with severe burns completed the BOQ sufficiently. The only statistically significant difference between the 102 responders and the 159 non-responders was the mean %TBSA burned; responders had a higher mean %TBSA burned (7.1% vs. 5.9%; *p* = 0.011) compared to non-responders (Appendix A).

### 3.2. Participant Characteristics

Children had a mean age of 2.8 years (SD 3.0) at the time of the burn injury and were on average 8.4 years old (SD 3.0) at the time of study (Table 1). Most of them were boys (n = 60; 58.8%) with a mean %TBSA burned of 7.1% (SD 6.4). More than a third of the children (n = 35; 34.3%) had at least one operation for their burns. The majority of burns were caused by scalds (n = 88; 86.3%). None of the children needed artificial ventilation.

Characteristics were compared between children with mild/intermediate burns and children with severe burns. Except for sex and age at burn, all characteristics differed significantly between the two groups. Children with mild/intermediate burns had a shorter mean LOS (5.9 days vs. 22.9 days), and a higher proportion had no surgeries (75.9% vs. 30.4%) compared to children with severe burns (Table 1).

### 3.3. Parent-Reported Pediatric Health-Related Quality of Life

Five to seven years post-burn, almost half of the parents (n = 47; 46.1%) reported that the general health of their child was excellent; more than a third (n = 36; 35.3%) found their child’s health to be very good; and the remaining parents scored their child’s general health as good (n = 15; 14.9%) or moderate (n = 3; 3.0%). None of the parents scored their child’s general health as bad. Almost all (n = 99; 97.0%) reported that their child’s general health was the same or better compared to before the burn injury.

Parents reported few problems in the different BOQ domains (Table 2). The three highest scores, and so the least problems (97.7–99.1%), were found for the domains of pain, physical function and sports, and upper extremity function. In total, 82 (81.2%) of the children had no problem in any of these domains. The three lowest scores were found for the domains of compliance with treatment, appearance, and satisfaction with current state (83.2–87.0%). For the domain compliance with treatment, a high proportion of parents reported that this was not applicable, resulting in a large % of missing values for this domain (51.0%). The BOQ domain school re-entry has a different scoring than the other 11 domains; an optimal score is above 50%. School re-entry scored at 69.0%, indicating that the children had relatively few problems with acceptance by peers and teachers and with doing their homework. Despite the relatively high scores and thus the low percentage of children with problems in a specific domain, in total, only two percent (n = 2) of the children reported no problems for all of the 12 domains. This indicates that the vast majority of all children encountered some HRQL-related problems. About one in four children (25.5%) reported problems in at least half of the domains.

### 3.4. Comparison of Subgroups

Many parents of children who had mild/intermediate burns scored their child’s general health as excellent (n = 34; 43.6%) or as very good (n = 29; 37.2%). The general health of children with severe burns was scored as excellent (n = 13; 56.5%) or very good (n = 7; 30.4%). The majority of the parents in both subgroups scored their children’s general health as unchanged 5–7 years after their burn injury compared to before their burn injury (mild/intermediate burns: 85.9% vs. severe burns: 81.8%; *p* = 0.713).

Both subgroups had high scores for the domains of pain (97.3–99.0), physical function and sports (98.8–99.2), and upper extremity function (99.0–99.2) (Table 2). These scores were not statistically significantly different (*p* = 0.075–0.743) between the two subgroups. The scores for the domains of appearance, parental concern, and school re-entry were significantly different between the subgroups. The scores of children with severe burns were significantly lower for appearance (71.5; *p* = 0.014) and parental concern (84.8; *p* = 0.021) than the scores of children with mild/intermediate burns (respectively, 85.0 and 94.1). In contrast, for school re-entry, children with severe burns scored significantly higher than children with mild/intermediate burns (81.0% versus 66.1%; *p* = 0.046), indicating that children with severe burns feel more positively about their acceptance by peers and teachers.

### 3.5. Predictive Factors of Parent-Reported Pediatric Health-Related Quality of Life Scores

Univariate predictive factors for the BOQ domains are shown in Table 3. No predictive factors were found for five domains: pain, upper extremity function, satisfaction with current state, compliance with treatment, and emotional health. For the other seven domains, the number of univariate associated factors (*p* < 0.100) varied between one and six factors, including %TBSA, LOS, upper limb burns, facial burns, %TBSA full-thickness, and number of surgeries.

Multivariate predictive factors are presented in Table 4. For the seven domains, several independent predictive factors were uncovered. The percentage of full-thickness burns was statistically significantly associated with three domains, namely physical function and sports (*p* < 0.001), appearance (*p* < 0.001), and parental concern (*p* < 0.001). A higher percentage of full-thickness burns was related to more problems in all of these domains. The number of surgeries was found to be associated with more itch problems (*p* < 0.001) but with fewer problems with school re-entry (*p* = 0.009). The length of hospital stay was found to be associated with more problems in the domains ‘physical function and sports’ (*p* = 0.011) and ‘transfers and mobility’ (*p* < 0.001). Facial burns were found to be associated with more problems in the domain of family disruption (*p* = 0.036).

## 4. Discussion

This study evaluated the parent-reported burn-specific HRQL of children 5–7 years after their burn injury. The vast majority of parents scored their children’s general health as good or better compared to the pre-injury level. However, no problems in any of the 12 BOQ domains were reported for only two percent of the children. For about one in four children, parents reported problems on at least half of the domains. The least problems were reported for the domains of ‘pain’, ‘physical function and sports’, and ‘upper extremity function’, whereas the most problems were relatively reported for the domains of ‘compliance to treatment’, ‘appearance’, and ‘satisfaction with current state’. We compared the outcomes of children with mild/intermediate burns to those of children with severe burns. Parents of children with severe burns gave significantly worse scores for their children’s ‘appearance’ and ‘parental concern’ domains. In contrast, significantly more problems with school re-entry were reported for children with mild/intermediate burns. Six factors, including higher %TBSA, increased LOS, upper limb burns, facial burns, higher %TBSA full-thickness burns, and number of surgeries, were found to predict worse outcomes on seven BOQ domains.

Our results are in line with the results of earlier research on the short- and longer-term consequences of burn injury using BOQ. A study by Sveen et al. (mean %TBSA burned 10.5%) also demonstrated that children who had suffered burns had a relatively good burn-specific HRQL ≥ 5 years after burn injury [22]. When comparing specific BOQ domains, some similarities and some differences are present; the lowest scores in both studies were reported for the domain ‘appearance’ (77 vs. 83), which seems to be a universal issue in children with burns, also in the long term [4,23]. Parents of children in both the study of Sveen et al. and our study reported low scores for the domain ‘emotional health’ (s89 and 92.4) [22]. In contrast, in our study, lower scores were found for the domain ‘compliance to treatment’ (83.2 vs. 90). Meyer et al. (mean TBSA 35.5%) reported more problems in all BOQ domains at up to 48 months after discharge compared to our subsample of children with severe burns [23]. Parents in that study reported most problems in the domains of ‘appearance’, ‘compliance to treatment’, ‘emotional health’, and ‘school re-entry’. In our study, scores for ‘school re-entry’ were relatively good. In line with the results reported by Meyer et al., in our study, children with severe burns also reported relatively more problems in the domains ‘appearance’ and ‘compliance to treatment’. In addition to our study, Warner et al. (mean TBSA 34.5%) reported that the recovery of children did not reach the score of 90 in any of the BOQ domains at up to 48 months after burn injury [24]. In the present study, children with severe burns had an average score of 90 or higher in six domains, namely ‘pain’, ‘physical function and sports’, ‘upper extremity function’, ‘transfer and mobility’, ‘family disruption’, and ‘emotional health’. The lower scores reported by Warner et al. are explained by the higher percentage of TBSA of their study patients (35% vs. 16%) and possibly the shorter time after burn injury compared to our study, as children’s recovery may keep on enhancing after 48 months post-burn. A study by van Baar et al. (mean TBSA not reported, 89% TBSA < 10%) reported that more than half of the children experienced some long-term consequences 24 months after burns, with issues mainly related to ‘itch’, ‘appearance’, ‘satisfaction with current state’, ‘emotional health’, and ‘parental concern’ [4]. In the present study, these domains also scored relatively low, indicating that issues in these domains persist over time. It is essential to ensure that attention is consistently directed toward these domains at all stages of follow-up care.

Some studies applied generic instruments to assess children’s long-term HRQL instead of burn-specific ones [18,20,25]. Generally, the results of those studies are in line with the results found in our study. For example, the study of Laitakari et al. showed that 5–9 years post-burn, the HRQL of children with burns (mean TBSA 9.5%) was good on almost every dimension of the 17D questionnaire, which is in line with our results for children with mild/intermediate burns [20]. Compared to other dimensions, more problems with the dimensions of sleeping, concentration, discomfort, and depression were reported. In our study, some of these themes, like ‘having nightmares’ and ‘feeling depressed’, are included in the ‘emotional health’ domain. The ‘emotional health’ domain scored relatively good (score 93/100) in our study, though it scored lower in comparison to many of the other domains. The results of a study by Maskell et al. showed that children with a mean burned TBSA of 23% had significantly more psychosocial problems compared to children without burns 7.3 years post-burn, as assessed using the Peadiatric Quality of Life Inventory [26]; the main difficulty was school functioning. In the present study, relatively good scores were found for children with severe burns in the ‘emotional health’ domain. The BOQ also includes the domain of ‘school re-entry’, which, in contrast to the study by Maskell et al., scored relatively well in our subset of children with severe burns. A possible explanation could be the difference in the mean percentage of TBSA burned (23% vs. 16%). Furthermore, in the Netherlands, returning back to school is an integrated part of burn aftercare in children with severe burns. This can possibly be reflected in the ‘school re-entry’ domain score. In a study by Spronk et al., the EQ-5D was used to assess the generic HRQL of children 5–7 years post-burn. The present study is a follow-up study on the study of Spronk et al., and the children included in our study are a subset (79%) of that study. However, this study employed a different outcome measurement instrument, namely the BOQ. Spronk et al. reported that the majority (76%) of the children did not experience any problems with the six EQ-5D domains [18]. However, in the present study, relatively more problems were reported, with only 2% of the children reporting no problems in any domain. These differences might be explained by the fact that our sample is a subsample of the sample used in Spronk et al. and/or by the sensitivity of the instrument used. It could be that parents of children with more problems were more inclined to participate in the follow-up study. Also, the BOQ might be more sensitive to burn-related problems than the EQ-5D, which could result in more problems being identified.

Overall, in our study, parents of children with mild/intermediate burns reported fewer problems than children with severe burns. Children with severe burns scored significantly lower on the ‘appearance’ domain. Children with severe burns are likely to have more scars and therefore have more problems with the ‘appearance’ domain. Also, the ‘parental concern’ domain was scored significantly lower by parents of children with severe burns. The domain of parental concern addresses worries or concerns related to the child’s full recovery, level of pain or suffering, and future health. Parents of children with severe burns possibly experienced a larger trauma and thereby more stress. In addition, their children have a higher chance of having extensive scars. Feelings of stress and concern can be amplified by constantly being reminded of the trauma through the scars.

It is remarkable that children with severe burns experience significantly fewer problems with the domain ‘school re-entry’. A previous study in the United States of America showed that children with severe burns (23%) rarely return to school without losing grades [25]. The amount of support children with severe burns receive might explain this difference. As part of the Dutch burn aftercare process, children with severe burns are offered to return to school under the supervision of a burn center pedagogical staff member. For children with mild/intermediate burns, this is provided in consultation with the child and their parents. This is considered a critical factor for facilitating an optimal return to school.

Previous evidence suggests that %TBSA burned, percentage full-thickness burns, facial burns, hand burns, LOS, and time since burn are strong predictors of a worse HRQL in children with burns [18,27,28]. In our study, the percentage of full-thickness burns was found to be a predicting factor for problems in the domains of ‘parental concern’, ‘physical function and sports’, and ‘appearance’. Full-thickness burns are usually treated surgically and often result in relatively poorer scars. Moreover, hospitalizations for such injuries are often prolonged and more intense for both the child and their parents, causing physical and mental challenges [29,30]. Facial burns and upper limb burns were found to be predictors for problems in the domain of ‘family disruption’. The number of surgeries was found to predict more itch problems, indicating that itch is a long-term issue, especially for children with multiple scars.

The clinical implications of our study are relatively straightforward. Most children recover well following burn injuries. However, a small subset does not. The quality of aftercare provided in our burn centers likely plays a significant role in these outcomes. Healthcare professionals can use the aforementioned factors for a worse HRQL to support the process of screening for the consequences of burn injuries. Additionally, we implemented a patient-reported outcome system, the Burn centers Outcomes Registry the Netherlands for children (BORN kids), which can be utilized to deliver targeted aftercare. BORN kids starts at discharge and is presented at 3, 6, 12, months post-discharge and thereafter annually. However, there is currently a lack of clear cutoff points for many of the measurement instruments included within BORN kids. Nevertheless, the information obtained can be applied to optimize aftercare in our specialized burn care centers.

### Strengths and Limitations

This study included some strengths and limitations. A strength is the relatively large sample size, which enabled us to study the results of specific subgroups of children with burns. Another strength is the inclusion of children with mild/intermediate burns, as most long-term studies on HRQL in children mainly focus on children with major burns. This study thereby provides new important insights. Also, the multicenter nature of this study was a strength, as it provides insights on national level. Another strength is the use of the BOQ, which is the most used instrument to assess pediatric HRQL in children with burns. In addition, this instrument has been validated in Dutch [16]. However, the application of the BOQ has certain limitations. It demonstrates lower sensitivity in assessing children with minor burns, as some questions are specifically designed for children with scars—an outcome not present in all cases of minor burns. Additionally, the BOQ was developed using existing published instruments rather than being directly informed by input from children themselves. Furthermore, parents completed the questionnaire, resulting in parent proxy data. If children complete the questionnaire on their own, the questions may be scored differently. Previous studies showed that scores were, in general, comparable, but some significant differences had been reported on the domains of ‘appearance’ and ‘family disruption’ [10]. The comparison of child-reported HRQL versus parent proxy data remains an interesting topic for future research. Finally, although our non-responder analyses only revealed a difference in one out of nine tested characteristics, we cannot exclude a response bias, for instance, one related to the socioeconomic status of respondents. As a result, it is possible that specific groups, for instance, parents with a low socioeconomic status, are less represented in our sample.

## 5. Conclusions

The majority of children with burns in our sample had a good HRQL 5–7 years post-burn. However, some children still experienced problems because of their burns, even 5–7 years after the injury. Children with severe burns scored substantially lower on the domains ‘appearance’ and ‘parental concern’, possibly indicating the need for more emotional support. Children with full-thickness burns and those who underwent surgery had a higher risk of a poorer long-term HRQL, especially in 5 out of 12 domains (‘physical function and sports’, ‘transfer and mobility’, ‘appearance’, ‘parental concern’, and ‘itch ‘). The results of this study can be used to inform children with burns and their families about the long-term implications of burns. In addition, healthcare professionals may use these insights to identify children at risk for worse long-term HRQL outcomes earlier, potentially leading to better HRQL outcomes.

## Figures and Tables

**Table 1:** Participant characteristics. Data are shown as (M, SD) = mean, standard deviation or as number (%).

Variable	Total (n = 102)	Mild/Intermediate Burns (n = 79)	Severe Burns (n = 23)	*p*-Difference *
Sex: boy (n, %)	60 (58.8%)	44 (55.7%)	16 (69.6%)	0.234
Age at survey (years, M, SD)	8.4 (3.0)	8.3 (3.2)	9.0 (2.4)	**0.006**
Age at burn (M, SD)	2.8 (3.0)	2.8 (3.1)	2.6 (2.6)	1.000
%TSBA burned (M, SD)	7.1 (6.4)	4.7 (2.6)	15.6 (8.2)	**<0.001**
% TSBA full-thickness (M, SD)	0.9 (2.6)	0.2 (0.8)	3.8 (4.8)	**<0.001**
Length of hospital stay (days, M, SD)	9.8 (13.6)	5.9 (5.8)	22.9 (22.4)	**<0.001**
Number of surgeries (n, %)				
0	67 (65.7%)	60 (75.9%)	7 (30.4%)	**<0.001**
1	29 (28.4%)	18 (22.8%)	11 (47.8%)	
>1	6 (5.9%)	1 (1.3%)	5 (21.8%)	
Reconstructive surgery (n,%)	3 (2.9%)	0 (0.0%)	3 (12.9%)	**0.001**
Etiology(%)				0.990
Scald	88 (86.3%)	68 (86.1%)	20 (87.0%)	
Flame	9 (8.8%)	7 (8.9%)	2 (8.7%)	
Other	5 (4.9%)	4 (5.1%)	1 (4.3%)	
Time since burn (years) (M, SD)	5.7 (0.6)	5.5 (0.3)	6.4 (0.8)	**<0.001**
Location of burn injury (n, %)				
Head/face/neck	43 (42.2%)	25 (31.6%)	18 (78.3%)	**<0.001**
Trunk	72 (70.6%)	51 (64.6%)	21 (91.3%)	**0.014**
Arm	53 (52.0%)	35 (44.3%)	18 (78.3%)	**0.004**
Hand	22 (21.6%)	13 (16.5%)	9 (39.1%)	**0.021**
Leg	32 (31.4%)	20 (25.3%)	12 (52.2%)	**0.015**
Feet	10 (9.8%)	7 (8.9%)	3 (13.0%)	0.555
Genitals	5 (4.9%)	4 (5.1%)	1 (4.3%)	0.889
Buttocks	6 (5.9%)	5 (6.3%)	1 (4.3%)	0.724

Note. Severe burns: children aged <10 years old at injury with >10% TBSA, children aged ≥10 years old at injury with >20% TBSA, children with >5% full thickness burns. * *p*-values in bold indicate statistically significant values.

**Table 2:** Parent-reported pediatric health-related quality of life scores in children 5–7 years post burn.

Burn Outcomes Questionnaire (BOQ) Domains ^1^	Total (n = 102) Mean Score (SD)	Mild/Intermediate Burns (n = 79) Mean Score (SD)	Severe Burns ^2^ (n = 23) Mean Score (SD)	*p*-Difference ^3^
Pain	97.7 (11.1)	97.3 (12.4)	99.0 (4.7)	0.702
Itch	91.0 (16.7)	91.6 (16.3)	88.9 (18.2)	0.411
Physical function and sports	98.4 (6.8)	99.2 (4.2)	95.8 (12.1)	0.075
Upper extremity function	99.1 (3.3)	99.2 (3.1)	99.0 (4.0)	0.743
Transfers and mobility	99.6 (2.9)	99.7 (1.7)	98.9 (5.2)	0.632
Compliance to treatment	83.2 (25.1)	81.8 (28.6)	87.0 (11.4)	0.526
Appearance	85.0 (24.9)	89.2 (20.4)	71.5 (32.9)	**0.014**
Satisfaction with current state	87.0 (20.7)	88.1 (20.0)	83.7 (22.6)	0.056
Family disruption	95.8 (11.7)	96.3 (11.0)	94.1 (14.0)	0.256
Parental concern	92.0 (18.9)	94.1 (16.7)	84.8 (24.1)	**0.021**
Emotional health	92.4 (11.4)	92.7 (11.3)	91.3 (11.6)	0.410
School re-entry	69.0 (20.4)	66.1 (18.1)	81.0 (26.2)	**0.046**

^1^ The best score is 100 for all domains, except for school re-entry; an optimal score for school re-entry is a score > 50. ^2^ Severe burns: >10% total body surface area (TBSA) burned if aged <10 years old at burn, >20% TBSA if aged ≥10 years old at burn, or more than 5% full-thickness burns. ^3^ *p*-values in bold indicate statistically significant values.

**Table 3:** Univariate regression analyses for long-term pediatric health-related quality of life.

	Burn Outcomes Questionnaire (BOQ) Domains (Regression Coefficient)											
	Pain	Itch	PF #	UE #	TM #	CO #	AP #	SF #	FD #	PC #	EH #	SR #
Variables												
Boy vs. girl	−2.643	−2.036	−1.897	−1.122	−0.750	7.125	−6.897	−1.528	−4.469	−5.179	−3.304	−5.952
Age at burn injury	0.139	0.155	−0.118	0.140	−0.073	1.013	−0.606	0.004	−0.579	0.246	−0.088	−0.735
Age at study	0.179	0.227	−0.169	0.130	−0.088	1.171	−0.921	−0.157	−0.727	−0.017	−0.191	−0.696
% TBSA	0.087	−0.359	−0.490 *	0.027	−0.207 *	−0.138	−0.847 *	−0.183	0.079	−0.492	0.090	0.764
% TBSA full-thickness	−0.032	−0.719	−1.541 *	−0.084	−0.612 *	−0.766	−3.760 *	−0.456	−0.448	−2.367 *	−0.191	0.332
Length of hospital stay	0.027	−0.140	−0.298 *	0.013	**−0.133**	−0.143	**0.543**	−0.085	−0.029	−0.302 *	0.027	0.132
Number of surgeries	−1.831	−5.953 *	−2.673 *	−0.034	−1.046 *	−1.341	−8.011 *	0.862	−1.536	−5.129 *	−0.157	4.930 *
Facial burn vs. other	0.809	−4.753	−1.636	−0.381	−0.510	2.351	−12.314 *	−4.590	−4.908 *	−6.596	−1.439	−1.840
Lower limb burn vs. other location	1.957	0.014	−0.704	1.190	−0.368	−5.876	5.304	−2.602	−0.262	5.142	2.646	−4.386
Upper limb burn vs. other location	0.491	−7.496 *	−0.751	−1.095	−0.194	−5.671	−14.539 *	−7.575	0.185	−6.251	−0.725	11.650
Scalds vs. other etiology	−0.409	2.338	2.181	−0.511	1.006	4.217	10.381	−0.735	3.855	0.925	1.822	0.159

# PF = physical function and sports; UE = upper extremity function; TM = transfer and mobility; CO = compliance with treatment; AP = appearance; SF = satisfaction with current state; FD = family disruption; PC = parental concern; EH = emotional health; SR = school re-entry. * Values in bold indicate statistically significant values (*p* < 0.05).

**Table 4:** Multivariate regression analyses for long-term pediatric health-related quality of life *.

	BOQ Domains (Regression Coefficient)						
	Itch	PF #	TM #	AP #	FD #	PC #	SR #
Variables							
Boy vs. girl	**-**	-	-	-	-	-	-
Age at burn injury	-	-	-	-	-	-	--
Age at study	-	-	-	-	-	-	-
% TBSA	-	-	-	-	-	-	-
% TBSA full-thickness	-	−0.820	-	−3.760	-	−2.367	-
Length of hospital stay	-	−0.172	−0.133	-	-	-	-
Number of surgeries	−5.953	-	-	-	-	-	4.930
Facial burn vs. other	-	-	-	-	−4.908	-	-
Lower limbs vs. other	-	-	-	-	-	-	-
Upper limbs vs. other	-	-	-	-	-	-	-
Scalds vs. other	-	-	-	-	-	-	-

# PF = physical function and sports; UE = upper extremity function; TM = transfer and mobility; CO = compliance with treatment; AP = appearance; SF = satisfaction with current state; FD = family disruption; PC = parental concern; EH = emotional health; SR = school. * Number of surgeries was highly correlated with %TBSA full-thickness, %TBSA, and length of hospital stay and was therefore not included in multivariate regression analysis.

## Data Availability

The data that support the findings of this study are available from Margriet van Baar (contact via data@burns.nl) upon reasonable request.

## Appendix A

**Table A1:** Baseline characteristics of responders versus non-responders.

Variable	Responders (n = 102)	Non-Responders (n = 159)	*p*- Difference*
Sex: boy, n(%)	60 (58.8%)	89 (56.0%)	0.650
Age at burn (M, SD)	2.8 (3.0)	3.2 (3.2)	0.175
%TSBA burned (M, SD)	7.1 (6.4)	5.9 (6.0)	**0.011**
% TSBA full-thickness (M, SD)	0.9 (2.6)	1.3 (2.9)	0.247
Length of hospital stay (M, SD)	9.8 (13.6)	8.2 (9.1)	0.184
Number of surgeries (M, SD)			0.386
0	67 (65.7%)	102 (64.2%)	
1	29 (28.4%)	40 (25.2%)	
>1	6 (5.9%)	17 (10.6%)	
Reconstructive surgery, n(%)	3 (3.0%)	9 (5.7%)	0.323
Artificial ventilation, n(%)	0 (0.0%)	2 (1.2%)	0.256
Etiology (%)			0.239
Scald	88 (86.3%)	124 (78.0%)	
Flame	9 (8.8%)	21 (13.2%)	
Other	5 (4.9%)	14 (8.8%)	

Data are shown as (M, SD) = mean, standard deviation or as number (%). Note. Severe burns: children aged <10 years old at injury with >10% TBSA, children aged ≥10 years old at injury with >20% TBSA, children with >5% full-thickness burns. * *p*-values in bold indicate statistically significant values.
